# Genomic characterization of invasive typhoidal and non-typhoidal *Salmonella* in southwestern Nigeria

**DOI:** 10.1371/journal.pntd.0010716

**Published:** 2022-08-26

**Authors:** Odion O. Ikhimiukor, Anderson O. Oaikhena, Ayorinde O. Afolayan, Abayomi Fadeyi, Aderemi Kehinde, Veronica O. Ogunleye, Aaron O. Aboderin, Oyinlola O. Oduyebo, Charles J. Elikwu, Erkison Ewomazino Odih, Ifeoluwa Komolafe, Silvia Argimón, Abiodun Egwuenu, Ini Adebiyi, Oluwadamilola A. Sadare, Tochi Okwor, Mihir Kekre, Anthony Underwood, Chikwe Ihekweazu, David M. Aanensen, Iruka N. Okeke

**Affiliations:** 1 Global Health Research Unit for the Genomic Surveillance of Antimicrobial Resistance, Department of Pharmaceutical Microbiology, Faculty of Pharmacy, University of Ibadan, Ibadan, Oyo State, Nigeria; 2 Department of Medical Microbiology and Parasitology, University of Ilorin, Ilorin, Kwara State, Nigeria; 3 Department of Medical Microbiology and Parasitology, University College Hospital, Ibadan, Oyo State, Nigeria; 4 Department of Medical Microbiology and Parasitology, Obafemi Awolowo University Teaching Hospitals Complex, Ile-Ife, Nigeria; 5 Department of Medical Microbiology and Parasitology, Faculty of Basic Medical Sciences, College of Medicine, University of Lagos, Lagos, Nigeria; 6 Department of Medical Microbiology, School of Basic Clinical Sciences, Benjamin Carson College of Health and Medical Sciences, Babcock University & Teaching Hospital, Ilishan-Remo, Ogun State, Nigeria; 7 Centre for Genomic Pathogen Surveillance, Big Data Institute, University of Oxford, Oxford, United Kingdom; 8 Nigeria Centre for Disease Control, Jabi, Abuja, Nigeria; Institut Pasteur, FRANCE

## Abstract

**Background:**

Salmonellosis causes significant morbidity and mortality in Africa. Information on lineages of invasive *Salmonella* circulating in Nigeria is sparse.

**Methods:**

*Salmonella enterica* isolated from blood (n = 60) and cerebrospinal fluid (CSF, n = 3) between 2016 and 2020 from five tertiary hospitals in southwest Nigeria were antimicrobial susceptibility-tested and Illumina-sequenced. Genomes were analysed using publicly-available bioinformatic tools.

**Results:**

Isolates and sequence types (STs) from blood were *S*. Typhi [ST1, n = 1 and ST2, n = 43] and invasive non-typhoidal *Salmonella* (iNTS) (*S*. *Enteritidis* [ST11, n = 7], *S*. Durham [ST10, n = 2], *S*. Rissen [ST8756, n = 2], *S*. Chester [ST2063, n = 1], *S*. Dublin [ST10, n = 1], *S*. Infantis [ST603, n = 1], *S*. Telelkebir [ST8757, n = 1] and *S*. Typhimurium [ST313, n = 1]). *S*. Typhi ST2 (n = 2) and *S*. Adabraka ST8757 (n = 1) were recovered from CSF. Most *S*. Typhi belonged to genotype 3.1.1 (n = 44), carried an IncY plasmid, had several antibiotic resistance genes (ARGs) including *bla*_TEM-1_ (n = 38), *aph(6)-Id* (n = 32), *tet(A)* (n = 33), *sul2* (n = 32), *dfrA14* (n = 30) as well as quinolone resistance-conferring gyrA_S83Y single-nucleotide polymorphisms (n = 37). All *S*. Enteritidis harboured *aph(3”)-Ib*, *bla*_TEM-1_, *catA1*, *dfrA7*, *sul1*, *sul2*, *tet(B)* genes, and a single ARG, *qnrB19*, was detected in *S*. Telelkebir. Typhoidal toxins *cdtB*, *pltA* and *pltB* were detected in *S*. Typhi, Rissen, Chester, and Telelkebir.

**Conclusion:**

Most invasive salmonelloses in southwest Nigeria are vaccine-preventable infections due to multidrug-resistant, West African dominant *S*. Typhi lineage 3.1.1. Invasive NTS serovars, including some harbouring typhoidal toxin or resistance genes, represented a third of the isolates emphasizing the need for better diagnosis and surveillance.

## Author summary

Whole genome sequencing of 63 invasive *Salmonella* from 5 tertiary hospitals in Nigeria revealed multiple serovars including a dominant antibiotic-resistance-gene harbouring *S*. Typhi 3.1.1 genotype comprising a gyrA_S83Y and IncY plasmid. We also report invasive non-typhoidal *Salmonella* harbouring typhoidal toxins.

## Introduction

*Salmonella* are a group of Gram negative, motile, facultative anaerobic rod-shaped bacteria belonging to the *Enterobacteriaceae* family. This genus consists of two known species, *Salmonella enterica* and *Salmonella bongori*. *S*. *enterica* are further distributed across six subspecies, of which the *S*. *enterica* subsp. *enterica* are most reported in infections involving homeotherm animals [1]. Furthermore, *S*. *enterica* subsp. *enterica* consists of over 1500 serovars with distinct antigenic specificity [2]. The human host-adapted *S*. *enterica* subsp. *enterica* serovars are usually associated with three marked clinical syndromes: *Salmonella enterica* subsp. *enterica* serovar Typhi cause typhoid fever, and the non-typhoidal *Salmonella* (NTS) cause gastroenteritis in immunocompetent persons but can cause bacteraemia in immunocompromised (including persons with advanced HIV disease, cases of severe malaria and malnutrition in children) [3,4]. *S*. Paratyphi A, B and C produce a syndrome similar to typhoid fever.

The public health impact of typhoidal and invasive non-typhoidal *Salmonella* infections is significant particularly in Africa and Asia where they have a great influence on morbidity and mortality [5,6]. For instance, an estimated 17.8 million cases of typhoid fever occur each year in low and middle-income countries (LMICs) [7]. An earlier estimate suggests that the burden of typhoid fever is >100 per 100000 individuals per annum in sub-Saharan Africa with an associated 1% mortality [8,9]. Furthermore, an estimated 26% (33,490 lives lost) of the annual global typhoid-related mortality is reported to occur in Africa [9]. The disease burden of typhoid in Nigeria is estimated at 364,791 typhoid cases resulting in 4,232 deaths (with 68% of deaths recorded in individuals under 15 years of age) as at 2016 [10], however population-based data are only just becoming available [11]. Globally, NTS is estimated to cause approximately 94 million cases of gastroenteritis per annum worldwide with a resultant mortality of 155,000 [12]. In immunocompromised cases of the disease (amongst HIV-positive adults), NTS is reported to cause a 20% case fatality (212,000 deaths) in western, central, eastern and southern Africa annually, while also being responsible for over 1 million cases of bloodstream infections in children with a case fatality of 18.1% (197,000 child mortality) [3,13,14].

Although available reports suggest infection with *Salmonella enterica* to be the most common cause of bloodstream infections in Africa [15], the incidence and microbiology of typhoidal and invasive non-typhoidal *Salmonella* (iNTS) is still poorly understood. Many regions on the continent have garnered little or no attention in the literature [7]. Blood culture-based surveillance represents the standard method for assessing the epidemiology and aetiology of bacterial invasive infections [16]. Limited surveillance of invasive *Salmonella* on the Africa continent is majorly due to financial, logistical, and infrastructural constraints for the institution and maintenance of blood culture-based surveillance systems in the region [7,8,16,17].

Such limitations not only obscure the true burden and prevalence of invasive *Salmonella* infections in resource-limited settings but also limit opportunity for genomic surveillance of this pathogen. For instance, despite the huge burden of typhoid infections in Nigeria, before the current study, only 131 of total (n = 4389) *Salmonella* genomes (all *S*. Typhi) from the country was available on Pathogenwatch (https://pathogen.watch/, containing all publicly available genomes to November 2020) [18], a web-based platform for surveillance of microbial genomes, all of which were collected on or before 2013 and most from only two centres [19]. Outside this study, no public *S*. Typhi genomes from Nigeria were uploaded between November 2020 and November 2021. Lack of genomic surveillance information of invasive *Salmonella* in resource-limited countries, including Nigeria, may deter interventions necessary to ameliorate this burden, such as the typhoid conjugate vaccines [8,17,19,20]. Hence, this report provides genomic characterization of 2016–2020 invasive *Salmonella* retrieved from tertiary hospitals enrolled into Nigeria’s Antimicrobial Surveillance Network coordinated by the Nigeria Centre for Disease Control (NCDC).

## Materials and methods

### Ethics statement

Isolates were obtained as part of the surveillance efforts in line with Nigeria’s national action plan. Surveillance began in 2019 and labs in the surveillance system were also requested to forward retrospective isolates that had been collected since 2016. Therefore, isolates included in this report were obtained from 2017 to 2020. Ethical approval for using them in research was obtained from the University of Ibadan/University College Hospital ethics committee (UI/EC/15/093). Patient consent was not obtained and the data were analysed anonymously.

### Isolate collection, identification and antimicrobial susceptibility testing

Tertiary hospitals located in southwest Nigeria and enrolled into the Nigeria Antimicrobial Surveillance Network provided cryopreserved isolates from blood and cerebrospinal fluid to the AMR National reference laboratory. The isolates were from retrospectively batched periods of 2016–2018 (retrospective isolates), 2019 and 2020. The national reference lab in partnership with the Global Health Research Unit for the Genomic Surveillance of Antimicrobial Resistance (GHRU-GSAR) conducted the re-identification of the isolates using the Gram-negative (GN) test kit on (Ref: 21341) on VITEK 2 systems (version 2.0, Marcy-l’Etoile, France, Biomérieux). Briefly, the cryopreserved isolates (at -80°C) are resuscitated before use for reidentification by subculturing onto Salmonella-Shigella Agar and incubated aerobically at 37°C. Isolated colonies from pure cultures are the streaked on Nutrient Agar (NA), incubated aerobically at 37°C. Isolated colonies on NA is then used to prepare inoculum for VITEK using GN cards. This test is based on forty-seven biochemical tests and a negative control. The cards contain wells with substrates for the different tests in dried form. The cards are inoculated with a saline suspension of the organisms before incubation. Upon incubation, biochemical reactions are read by the machine and recorded as positive or negative. A bionumber which is based upon the combination of different test results is then generated. The bionumber is compared to VITEK 2 robust database to match the organism and this is used to identify the organism. An added step for confirming identity of VITEK-identified isolates utilized whole-genome sequencing of all isolates. Antimicrobial susceptibility testing was done using VITEK AST N280 test kit (Ref: 413432). N280 Cards are incubated within the VITEK 2 compact upon inoculation with appropriate saline suspension of test organism. The minimum inhibitory concentration (MIC) is recorded as the highest concentration of an antibiotic for which no bacterial growth is observed. The MICs were interpreted as either resistant, intermediate or susceptible in accordance to CLSI standards [21].

### DNA extraction and library preparation

The isolates were processed for the extraction of genomic DNA using Wizard DNA extraction kit (Promega; Wisconsin, USA) following manufacturer’s instructions. The extracted DNA was quantified on a Qubit fluorometer (Invitrogen; California, USA) using dsDNA Broad Range quantification assay. Double-stranded DNA libraries were prepared using the Covaris LC220 for fragmentation, and NEBNext Ultra II FS DNA library kit for Illumina with 384-unique indexes (New England Biolabs, Massachusetts, USA; Cat. No: E6617L). Libraries were sequenced on an Illumina HiSeq X10 (Illumina, California, USA).

### Genome assembly

Generated sequence reads from Illumina runs were *de novo* assembled following GHRU protocols (https://gitlab.com/cgps/ghru/pipelines/dsl2/pipelines/assembly) using a Nextflow workflow which inclusively comprises of adapter trimming (trimmomatic v0.38), contamination detection (ConFindr v0.7.2), assembly (SPAdes v3.12.0), Quality Control (multiqc v1.7, qualifyr v1.4.4) and Bactinspector (v 0.1.3).

### Sequence typing of *Salmonella* genomes

Sequence reads were deposited in the *Salmonella* database on EnteroBase [22]. Multi-locus sequence types (MLST) for the isolates were determined and core-genome MLST calculated. Evolutionary relationship based on cgMLST of all *S*. Typhi of human origin from Africa deposited in Enterobase were determined [22]. The *Salmonella* genome assemblies were analysed using the *Salmonella* In-Silico Typing Resource (SISTR) for the prediction of serovars and serogroups (https://github.com/phac-nml/sistr_cmd). Genomes belonging to *S*. Typhi were loaded unto Pathogenwatch for the prediction of their genotypes [18].

### Identification of AMR, plasmids, virulence genes and *Salmonella* pathogenicity islands

Determinants of AMR, virulence and plasmid replicons were identified following GHRU protocols (https://gitlab.com/cgps/ghru/pipelines). Prediction of *Salmonella* pathogenicity islands (SPIs) in the genomes was done by mapping raw reads to SPIs database (https://bitbucket.org/genomicepidemiology/spifinder_db)

### Single Nucleotide Polymorphism (SNP) calling and phylogeny

The sequence reads of the *S*. Typhi and *S*. Enteritidis genomes from our study were mapped to NCBI reference sequence, *Salmonella* enterica subsp. enterica serovar Typhi strain H12ESR00755-001A (assembly accession: GCF_001362195.2) and *Salmonella* enterica subsp. enterica serovar Enteritidis strain 18569 (assembly accession: GCF_000335875.2), respectively, to determine evolutionary relationship amongst the strains following GHRU nextflow SNP phylogeny protocols (https://gitlab.com/cgps/ghru/pipelines/snp_phylogeny). Briefly, reads were trimmed (trimmomatic v0.38) and mapped to the reference genomes described above using bwa mem (v0.7.17) and variants were called and filtered using bcftools (v1.9). A pseudoalignment with the reference was used to generate a maximum likelihood tree using iqtree (v1.6.8) [23]. SNP distances between the genome pairs were calculated using snp-dists v.0.8.2 (https://github.com/tseemann/snp-dists) on the pseudo-genome alignment.

## Results

### Invasive *Salmonella* from sentinel hospitals from Nigeria’s AMR surveillance network

Using the VITEK system for bacterial identification described above, a total of 69 isolates retrieved from patients from five (n = 5) sentinel hospitals were identified as *Salmonella* spp., at the reference laboratory. However, results from whole-genome sequencing confirmed n = 61 of these to be *Salmonella enterica*. In addition, two other isolates from our surveillance collection initially identified as *Escherichia coli* and *Acinetobacter baumanii* using VITEK were subsequently identified as *Salmonella enterica* using WGS.

**Fig 1:**
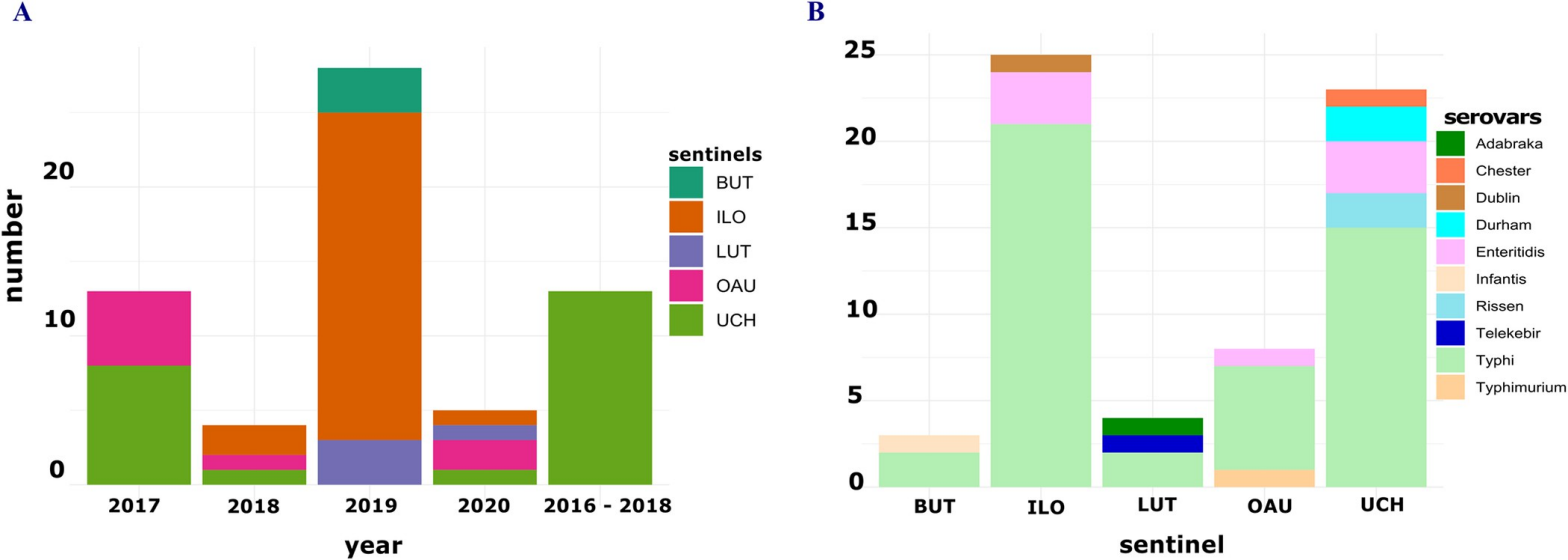
Epidemiological information showing. (A) number of Salmonella isolated received from the different sentinel hospitals at different years, and (B) Number of different *Salmonella* serotypes received from the different sentinel hospitals.

In the sixty-three (63) WGS-confirmed invasive *Salmonella* isolate genomes, the average number of contigs was 58 and N50 values ranged from 172132bp to 731013bp (average 246872 bp). The G+C (%) content of the genomes ranged from 51.86% - 52.37% (average 52.10%) (S1 Table). The isolates were retrieved from blood (n = 60) and cerebrospinal fluid (n = 3). The sending sentinel hospitals include: University of Ilorin Teaching Hospital (ILO, Ilorin, Kwara State, n = 25), University College Hospital, Ibadan (UCH, Ibadan, Oyo State, n = 23), Obafemi Awolowo University Teaching Hospital, Ile-Ife (OAU, Ile-Ife, Osun State, n = 8), Lagos University Teaching Hospital (LUT, Idi-Araba, Lagos State, n = 4) and Babcock University Teaching Hospital (BUT, Ilishan-Remo, Ogun State, n = 3) (Fig 1A). Majority of the isolates were retrieved in 2019 from ILO (n = 22). Thirteen isolates had no year specified metadata but were retrospective isolates retrieved between 2016 and 2018 (Fig 1A). The hospitals are all in the southwestern part of Nigeria with ILO just north of the South-West geopolitical zone and all the rest within it. All the *Salmonella* isolates from cerebrospinal fluid were obtained from LUT.

### Distribution of *Salmonella enterica* subsp. *enterica serovars* across sentinel hospitals

All the *Salmonella enterica* isolates belonged to the subspecies *enterica* but differed by serotype with a total of 10 serovars detected. They include Typhi (n = 46), Enteritidis (n = 7), Durham (n = 2), Rissen (n = 2), Adabraka (n = 1), Chester (n = 1), Dublin (n = 1), Infantis (n = 1), Telelkebir (n = 1), Typhimurium (n = 1). Three *Salmonella enterica* isolates belonging to serovars Adabraka (n = 1) and Typhi (n = 2) were retrieved from cerebrospinal fluid from LUT. All other *Salmonella* serovars were retrieved by blood culture at the respective sentinel sites (S1 Table). *Salmonella* Typhi and iNTS were recovered from all sentinel sites, with iNTS being much less frequently recovered (Fig 1B).

### Sequence types, genotypes, and nucleotide polymorphisms

*Salmonella* sequence-typing based on Achtman’s MLST scheme [24] identified two *S*. Typhi Sequence Types (STs) (ST1, n = 1 and ST2, n = 45). There were nine different iNTS STs. These included previously reported STs from invasive infections: *S* Enteritidis ST11 (n = 7) and *S* Typhimurium ST313 (n = 1), which are repeatedly reported from Africa. Other iNTS were *S*. Dublin (ST10), *S*. Infantis (ST603), *S*. Durham (ST2010), *S*. Chester (ST2063), *S*. Telelkebir (ST2222). Two novel STs belonging to *S*. Rissen and *S*. Adabraka were curated and designated STs 8756 and 8757 respectively by EnteroBase.

**Fig 2:**
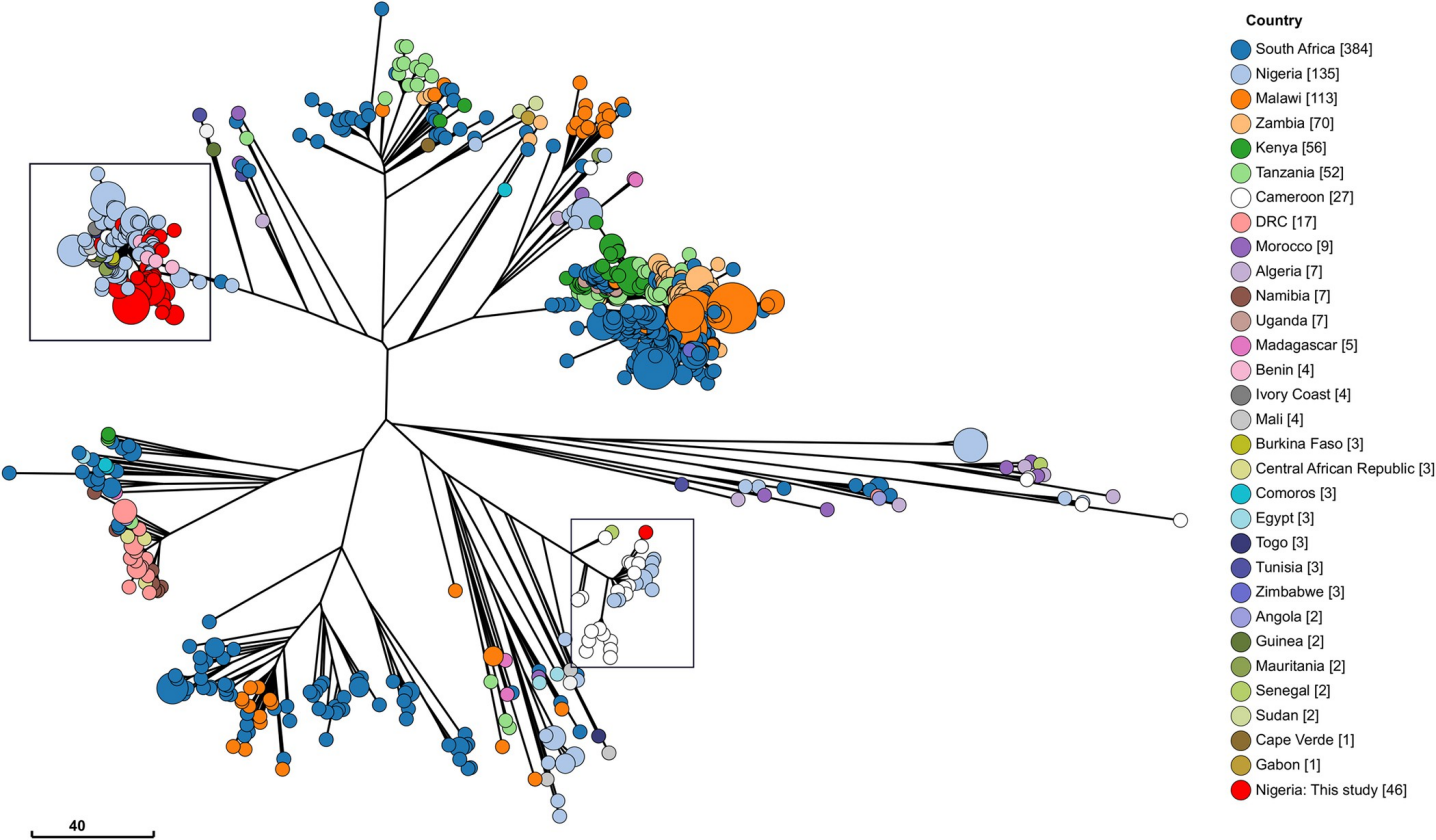
Grape tree showing core genome MLST of *S*. Typhi from human sources in Africa, deposited in the EnteroBase database. Red leaf labels are genomes from this study.

**Fig 3:**
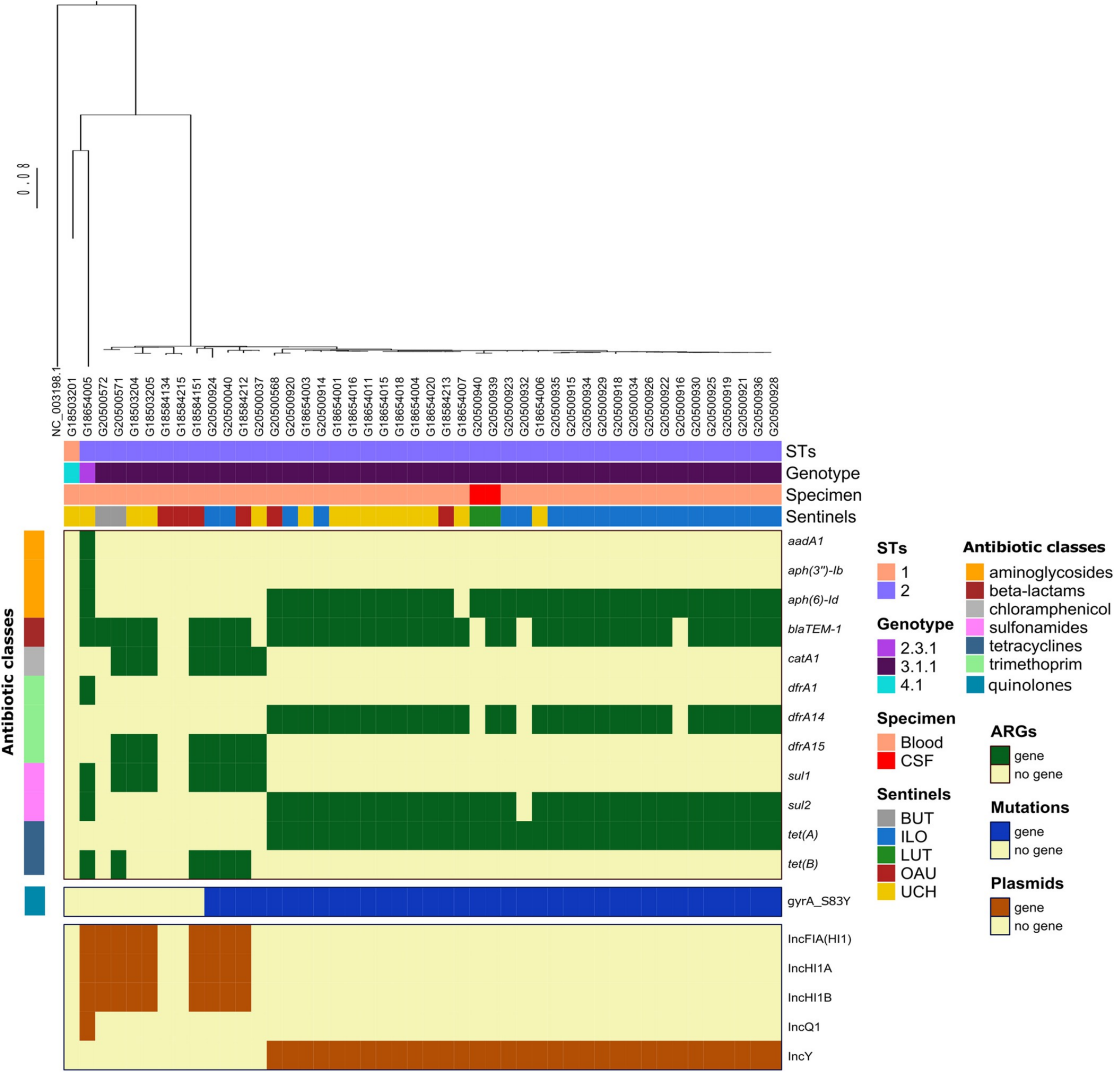
SNP-phylogeny based tree and gene presence/absence showing the genomic profile of *Salmonella* Typhi genomes retrieved from 5 sentinel laboratories in Nigeria. ARGs: antibiotic resistance genes, STs: Sequence types.

To further place our *S*. Typhi genomes in a wider context, we performed cgMLST analysis based on differences in core genomes of our strains and all *S*. Typhi from human sources in Africa deposited in EnteroBase (n = 980) (Fig 2). All genomes included in this study had similar core genome allelic differences at HC400, whereas at HC200 genomes from this study had similar allelic profile with 98.06% (n = 961) of the genomes in the population. Genomes accounting for the difference in cluster numbers in the population at HC400 were from Nigeria (n = 9, ~0.92%), Cameroon (n = 4, ~0.41%) Algeria (n = 3, ~0.3%) Morocco (n = 2, 0.2%) and Senegal (n = 1, 0.1%). Generally, *S*. Typhi genomes from this study clustered with others from West Africa, including Nigeria, Cameroon, Togo, Mauritania, Mali, Burkina Faso, Guinea, Benin, and Ivory Coast, emphasizing further on their endemicity in the West Africa region (Fig 2). Further, based on *S*. Typhi genotyping scheme, we observed that the isolate of *S*. Typhi ST1 belonged to genotype 4.1 (UCH), whereas genotypes 2.3.1 (n = 1, UCH) and 3.1.1 (n = 44) were *S*. Typhi ST2 isolates. In addition, *S*. Typhi genomes from CSF (n = 2) belonged to the 3.1.1 genotype (Fig 3).

**Fig 4:**
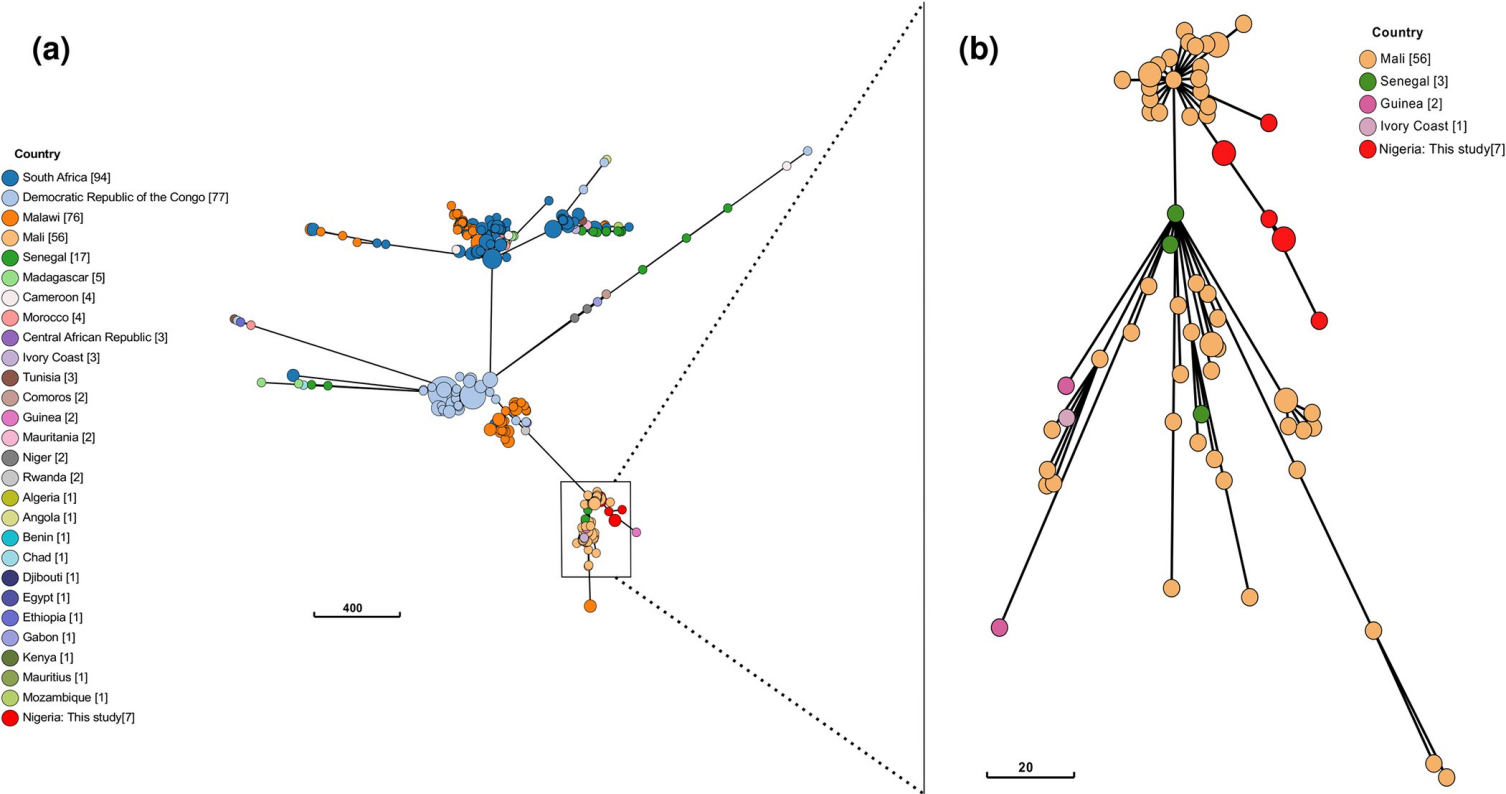
Grape tree showing core genome MLST of *S*. Enteritidis from human sources in Africa, deposited in the EnteroBase database. Red leaf labels are genomes from this study.

We compared sequence diversity of our *S*. Enteritidis genomes to *S*. Enteritidis collection from Africa described by Feasey et al. [25]. This collection included over 360 genomes from 27 countries in Africa, majority of which were from South Africa (n = 94), Democratic Republic of Congo (n = 77) and Malawi (n = 76) (Fig 4A). There were no genomes from Nigeria in this collection, except those from this study (Fig 4A). We observed our genomes clustered with other West Africa genomes from Mali, Senegal, Guinea and Ivory Coast (Fig 4B). All the genomes in this cluster belong to Feasey et al. [25] hierBAPS cluster 2 described as the multidrug resistant West African epidemic clade associated with human invasive infections and phenotypic and genotypic resistance to ≥ 1 antimicrobial class. In concordance with the study of Nikiema et al. [26], and as similarly observed with our genomes, members of this clade have similar core genome allelic profiles at HC100. To further investigate the genetic relatedness of the genomes in this study, we determined pairwise SNP differences among the genomes. We observed that the three *S*. Enteritidis isolates from ILO (n = 3) were near identical having pairwise SNP range from 0 to 1 (S4 Table).

### Antimicrobial susceptibility profiles, antimicrobial resistance determinants and plasmids replicons in TS and NTS

Antimicrobial susceptibility testing revealed majority of the *S*. Typhi to be resistant to sulphamethoxazole/trimethoprim (SXT) and ampicillin (n = 41 each) and nalidixic acid (n = 36), of which three were ciprofloxacin non-susceptible, according to CLSI (2021) criteria. (S2 Table). While not relevant to the antimicrobial chemotherapy of invasive infections, resistance to nitrofurantoin was identified in the n = 2 *S*. Typhi isolated from CSF and in n = 9 isolates from blood with the highest MICs (128 μg/mL) seen in the CSF isolates only. Resistance to cephalosporins, cefuroxime and cefuroxime axetil was observed in *S*. Typhi 3.1.1 from UCH.

The single *S*. Typhi 2.3.1 isolate was resistant to ampicillin and SXT whereas no phenotypic resistance was observed with *S*. Typhi 4.1. All *S*. Enteritidis and *S*. Typhimurium were resistant to ampicillin and sulphamethoxazole/trimethoprim. Asides Telelkebir harbouring resistance to nalidixic acid and ciprofloxacin, other NTS were either susceptible or intermediately resistant to other antimicrobials (S2 Table). For example, *S*. Adabraka, Dublin and Telelkebir were intermediately resistant to cefuroxime axetil. (S2 Table and microreact link for antimicrobial susceptibility testing: https://microreact.org/project/ahQ3Yb64nsbnhHMzz3WQn9-genomic-epidemiology-of-invasive-salmonella-in-southwestern-nigeria-ast-data)

A combined total of 14 acquired antimicrobial resistance genes (ARGs) conferring resistance to drugs within seven antibiotic classes were detected amongst the genomes. Amongst the *S*. Typhi genomes, n = 36/46 harboured at least one ARG conferring reduced susceptibility to 5 antibiotic classes, with n = 41 harbouring a sulphonamide resistance gene [*sul1* = 9/46, *sul2* = 33/46)] and n = 39 each harbouring a beta-lactam (*bla*_TEM-1_), tetracyclines (*tetA*, n = 33 and *tetB*, n = 6), and trimethoprim resistance determinant (*dfrA1*, [n = 1], *dfrA15* [n = 8] and *dfrA14* [n = 30]). In addition, chloramphenicol resistance genes, *catA1*, were also detected in the genomes (n = 8). Point mutations identified among the sequenced *S*. Typhi isolates were those associated with the quinolone resistance determining region (QRDR), gyrA_S83Y SNPs (n = 37), which mediate resistance to fluoroquinolones (Fig 3). Furthermore, n = 45 of the *S*. Typhi genomes had at least one plasmid predicted to occur in each genome. Majority (n = 33) possessed an IncY plasmid replicon, plasmid replicons IncFIA_HI1, IncHIA and IncHIB were respectively detected in n = 9 of *S*. Typhi genomes whereas one isolate harboured an IncQ plasmid replicon (Fig 3).

**Fig 5:**
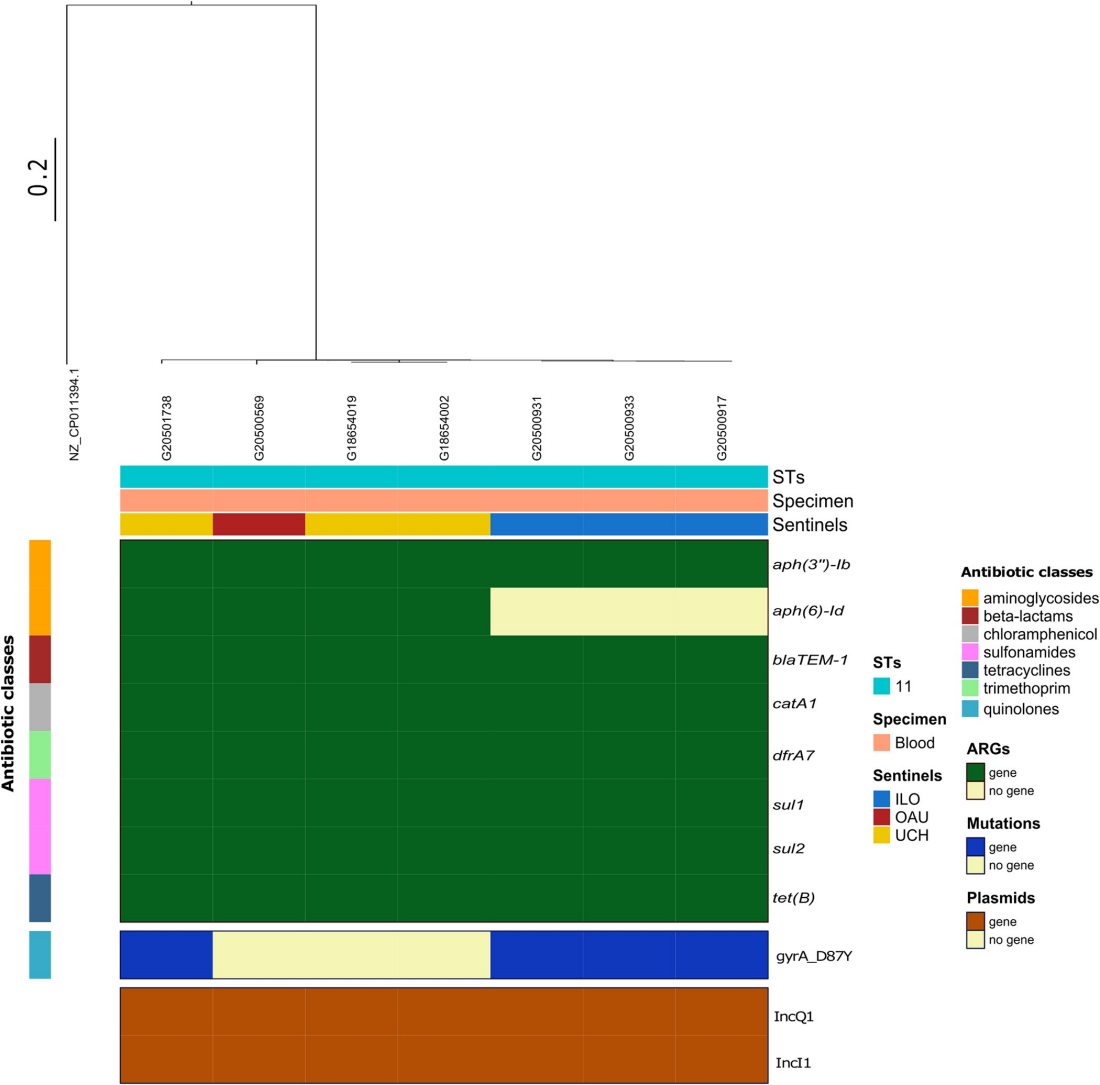
SNP-phylogeny based tree and gene presence/absence map showing the genomic profile of *Salmonella* Enteritidis retrieved from 3 sentinel laboratories in Nigeria. ARGs: antibiotic resistance genes, STs: Sequence types.

**Fig 6:**
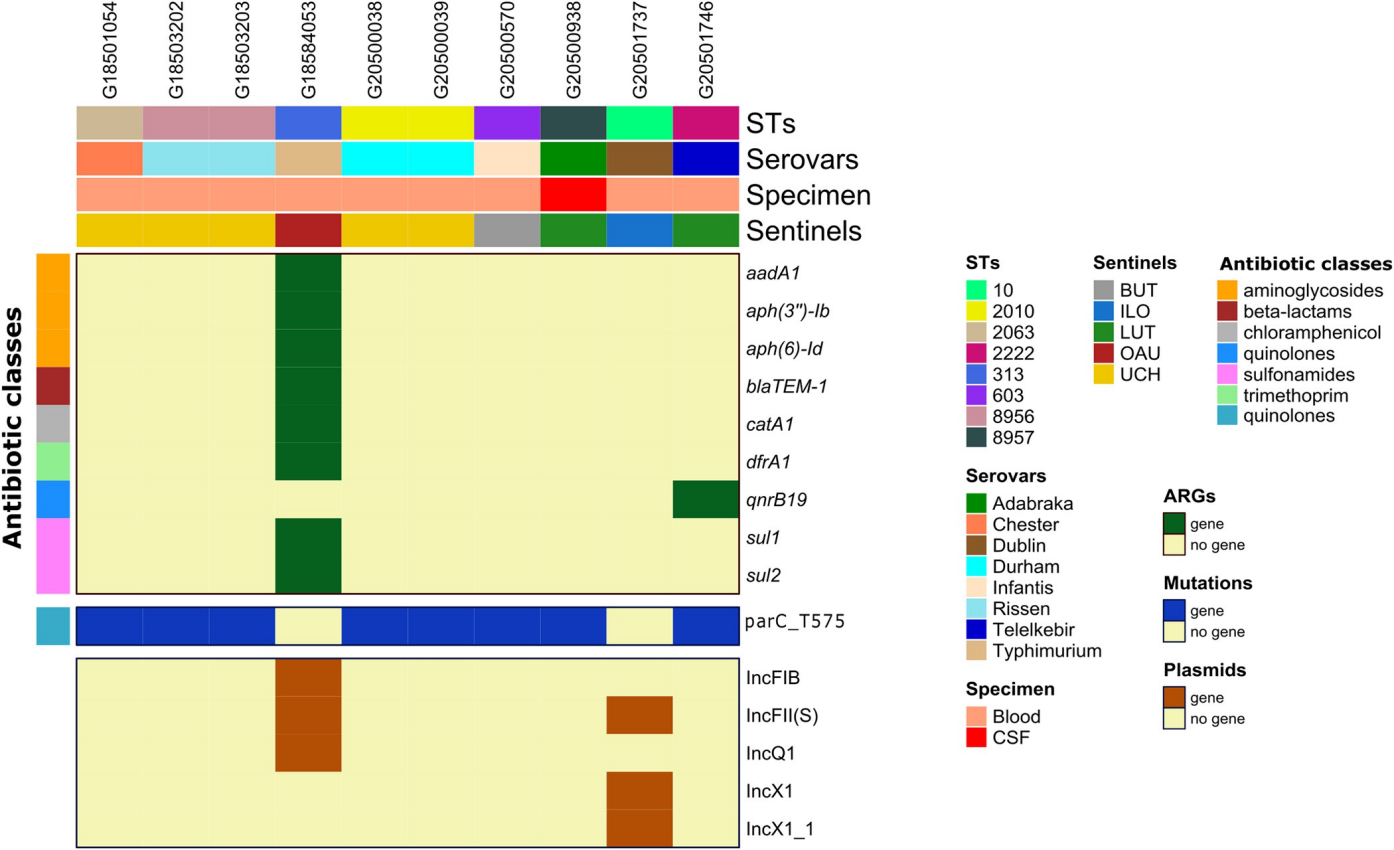
Gene presence/absence map showing the genomic profile of non-typhoidal *Salmonella* retrieved from 5 sentinel laboratories in Nigeria. ARGs: antibiotic resistance genes, STs: Sequence types.

For the iNTS, *S*. Enteritidis genomes possessed at least one ARG to six antibiotic classes. All Isolates of this serotype harboured *aph(3”)-Ib*, *bla*_TEM-1_, *catA1*, *dfrA7*, *sul1*, *sul2*, *tet(B)* genes, and only differed in the absence/presence of *aph(6)-Id* (n = 4) (Fig 5). In tandem, *S*. Typhimurium harbour ARGs [*aadA1*, *aph(3”)-Ib*, *aph(6)-Id*, *bla*_TEM-1_, *catA1*, *dfrA1*, *sul1*, *sul2*] encoding resistance to 5 antibiotic classes (Fig 6). The only occurring quinolone resistance gene among isolates in this study, *qnrB19*, was detected in *S*. Telelkebir. No ARGs were detected in *Salmonella* serovars Chester, Rissen, Durham, Infantis, Adabraka and Dublin. Antimicrobial point mutations identified among iNTS were associated with *gyrA* and *parC* gene regions (Fig 6). The quinolone resistance conferring gyrA_D87Y SNPs were identified only amongst *S*. Enteritidis (ILO, n = 3 and UCH, n = 1), whereas the parC_T57S mutations were detected in all iNTS except *S*. Enteritidis and *S*. Typhimurium. Plasmids were predicted to occur only in *S*. Dublin [IncFII(S), IncX1 and IncX1_1], *S*. Enteritidis (IncI1 and IncQ1), *S*. Typhimurium [IncFIB, IncFII(S) and IncQ1] among the iNTS.

### Predominant IncY + gyrA_S83Y + *tetA* harbouring *S*. Typhi 3.1.1 in Nigeria

We observe that all *S*. Typhi of the 3.1.1 lineage harbouring an IncY plasmid replicon (n = 33/46) similarly possessed the gyrA_S83Y chromosomal gene mutation and harboured a *tetA* gene. Although other antimicrobial resistance genes were seen at slightly lower numbers (n = 32/33 for *sul2* and *aph(6)-*Id and n = 30/33 for *bla*_TEM-1_ and *dfrA14*), the IncY+gyrA_S83Y+*tetA* in *S*. Typhi 3.1.1 phenomenon was observed to occur in all the sentinel hospitals in this study. Additionally, maximum pairwise SNP distance between the variants in this cluster was 23. This is approximately twice as less of what was determined in *S*. Typhi 3.1.1 outside this cluster (n = 47), thereby, emphasizing clonality within this cluster.

### *Salmonella* virulence determinants and predicted pathogenicity islands

The isolates possessed a plethora of virulence determinants (S2 Table). Among the *S*. Typhi genomes, a total of 98 virulence determinants were detected, and 97 of these were conserved within members of this serovar (with the exception of *pipB2* gene in an *S*. Typhi 3.1.1 from UCH).

A total of 106 virulence genes were detected among the *S*. Enteritidis genomes, 104 of these were conserved within these genomes, with 2 strains from UCH lacking either a *Salmonella* secreted protein H (*sspH*) or secretion system effector I (*sseI*). A total of 122 virulence genes were detected in the iNTS genomes, and n = 86 of these were conserved in all iNTS genomes. For instance, the iNTS possessed genes encoding (i) Adherence; such as *agf*–thin aggregative fimbrae or curli (*csgABCDEGF*), *misl*–an autotransporter protein, *pef*—plasmid-encoded fimbrae (present only in *S*. Typhimurium) *ratB* (carried by iNTS strains harbouring CS54 islands), *shdA* (only found in *S*. Infantis), *sinH* (detected in all NTS except *S*. Enteritidis) and Type 1 fimbrae (*fimCDFHI*) (ii) Stress adaptation; *sodCI*–superoxide dismutase (detected in iNTS serovars except Durham, Chester and Rissen and Infantis and Telelkebir), *sopA* (not detected in *S*. Infantis) (iii) Nutritional/metabolic factor (*mgtBC*, present in all strains) (iii) Antimicrobial activity/competitive advantage; such as macrophage inducible genes (*mig-14*, present in all strains) and (iv) Enterotoxin; T3SS effectors–*spvBC* (in *S*. Typhimurium, Enteritidis and Dublin), *avrA* (in all iNTS except *S*. Dublin) and Typhoidal toxin—*cdtB* (present in *S*. Durham, *S*. Telelkebir and *S*. Chester).

Since the *cdtB* are reported to be co-located with other cytolethal distending toxins (*cdt*), pertussis-like toxins A (*pltA*) and B (*pltB*), on same pathogenicity islet [27], we ran a blast search of our strains for the presence of *pltA* and *pltB*. The nucleotide sequences were extracted from the virulence factor database (VFDB) and used as a local database for a blast search against our iNTS genomes. Our results reveal high similarity (100% coverage and ≥ 96.62% identity) with *cdtB*, *pltA* and *pltB* genes in the iNTS genomes (*S*. Chester, *S*. Durham and *S*. Telelkebir).

**Fig 7:**
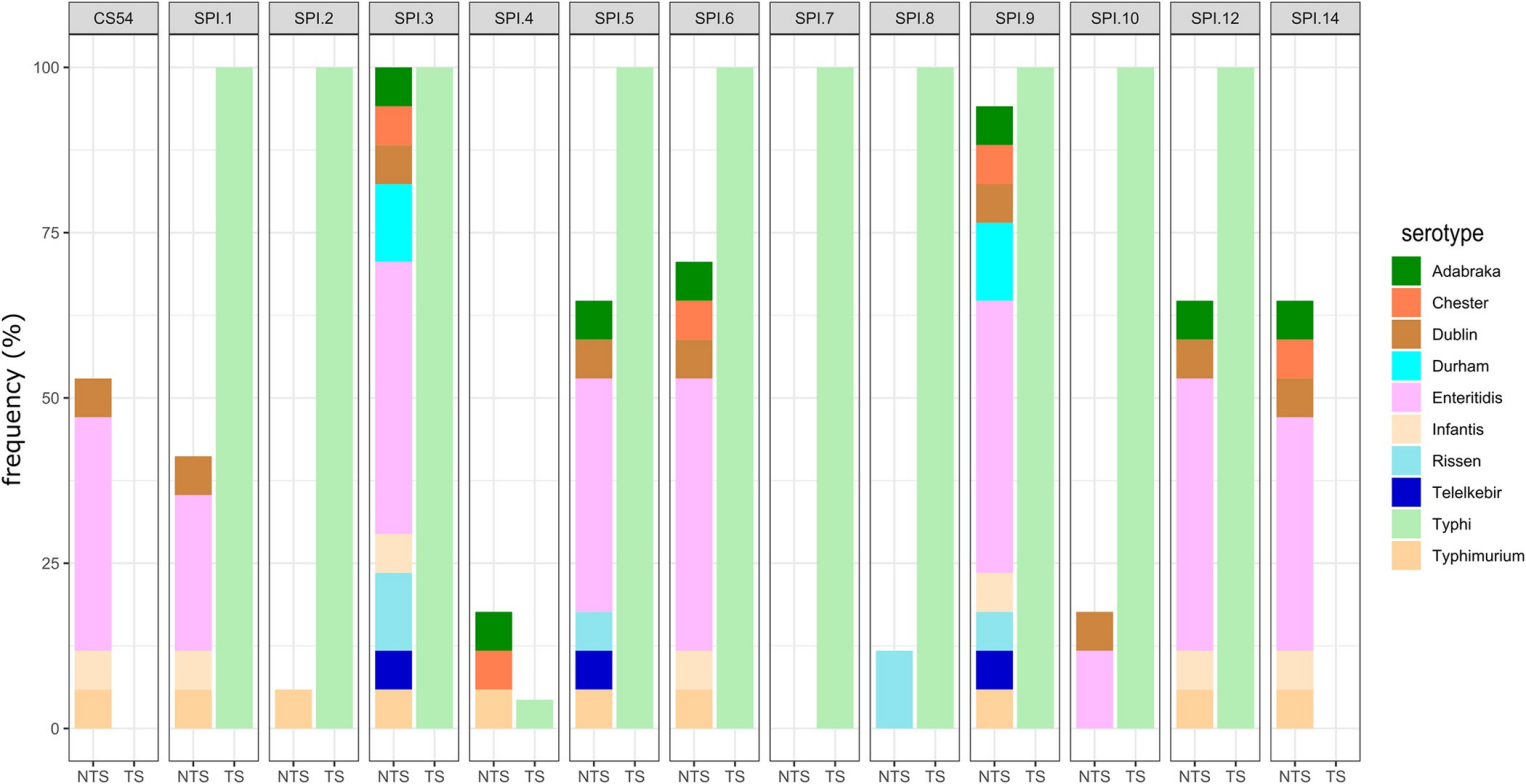
Frequency of occurrence of *Salmonella* pathogenicity island in TS (Typhoidal Salmonella) and NTS (Non-typhoidal Salmonella) in this study.

Eleven and twelve *Salmonella* pathogenicity islands (SPIs) were predicted in *S*. Typhi and iNTS genomes, respectively (Fig 7). All *S*. Typhi were predicted to have 11 SPIs, i.e., SPI-1, SPI-2, SPI-3, SPI-4, SPI-5, SPI-6, SPI-7, SPI-8, SPI-9, SPI-10 and SPI-12. However, SPI-4 was predicted to occur only in *S*. Typhi lineages 2.3.1 and 4.1. In contrast to *S*. Typhi, only SPI-3 was predicted to occur in all the iNTS genomes. Certain SPIs were shown to be associated with members of certain serovars. For instance, SPI-2 and SPI-8 were detected only in *S*. Typhimurium and *S*. Rissen, respectively. Other pathogenicity islands were detected in this study (Fig 3), such as SPI-4 (*S*. Adabraka, Chester, Typhimurium), SPI-6 (all NTS except *S*. Durham, Rissen and Telelkebir), SPI-12 (all NTS except S. Chester, Durham, Rissen and Telelkebir) and CS54_island was detected in *S*. Dublin, *S*. Typhimurium, *S*. Infantis and *S*. Enteritidis (n = 6).

## Discussion

In this report we present the outcome of genomic characterization of invasive *Salmonella* infections from AMR surveillance in sentinel hospitals in Nigeria. The genomic characterization of invasive *Salmonella* isolates in this study was possible because these hospitals perform blood culture and are enrolled in Nigeria’s new antimicrobial resistance surveillance system, which offers genomic services at the National Reference Laboratory level [28]. Nonetheless, blood culture is available at very few institutions in Nigeria, a limitation still prevalent in many African settings that impacts the genomic surveillance of invasive *Salmonella*. [8,16,29]. Moreover, these sentinels perform very few blood cultures so that the isolates studied here represent a very small proportion of circulating strains.

Using WGS and bioinformatic analytics, we were able to determine prevalent serotypes and dominant genotypes of invasive *Salmonella* infections. Most of the *Salmonella* isolates cultured from blood were *S*. Typhi, as has been previously reported from different parts of Nigeria, including Ibadan [30–32], even though iNTS may predominate in some other African settings [4,33]. While our data are few, the predominance of Typhi at all sites points to a significant burden of severe disease that could be averted if Typhoid Conjugate Vaccines were deployed in Nigeria. Out of a total of 10 *S*. Typhi genotypes recorded from Nigeria in Pathogenwatch, three were identified in this study. The *S*. Typhi genotype 3.1.1 we report was similarly common in the Nigeria cluster on Pathogenwatch (n = 87/131). As in our study, this cluster possessed similar prevalence of genetic determinants of beta-lactam (*bla*_TEM-1_−83.90%) resistance, indicating that these determinants are well-conserved in the genotype. However, prevalence of other AMR genetic determinants from this lineage such as *catA1*, *sul1*, *sul2*, *dfrA14*, *dfrA15*, *tetA*, *tetB* were similar with what is reported from this study, but at different rates.

This multidrug-resistance gene-encoding *S*. Typhi 3.1.1 is shown to be one of the broadest lineages in sub-Saharan Africa and endemic in the West Africa region [19,34,35]. This *S*. Typhi genotype is frequently reported to be multidrug and ciprofloxacin resistant [29,31]. All S. Typhi 3.1.1 (except one from OAU) harbouring quinolone-conferring SNPs in *gyrA* showed phenotypic resistance to nalidixic acid. Additionally, we observed that the *S*. Typhi genotype 3.1.1 clone variants harboured an IncY + gyrA_S83Y + *tetA* genes. The Pathogenwatch database includes three *S*. Typhi 3.1.1 strains isolated from blood samples in 2013, in Abuja, north-central capital of Nigeria with similar clonal characteristics (having same genotype, plasmid replicon, chromosomal QRDR and antimicrobial resistance gene, *tetA*) [19]. Outside Nigeria, this lineage has also been identified in the United Kingdom (accession: SRR7165434, SRR5585020) [32]. Our data suggest that this resistant sub-lineage is predominant in our setting and should be sought elsewhere in Nigeria and the region. In addition, long read sequencing to unveil the carriage of the IncY plasmids would be potentially vital to understanding the success of this lineage in Nigeria.

*S*. Enteritidis were the most frequently recovered iNTS in our study and sent from three sentinel hospitals. This outcome contrasts with earlier reports of *S*. Typhimurium ST313 as a predominant serotype in eastern and southern Africa, but also present across the continent, including Nigeria [15,36,37], but it is concordant with more recent reports describing *S*. Enteritidis in higher proportions in invasive infections in The Gambia [38]. Several of the *S*. Enteritidis in our study were multidrug resistant (resistant to ampicillin, SXT, nalidixic acid) and belong to the West African epidemic clade previously described [25]. This multidrug resistant clone has also been reported in bacteraemia in other parts of Africa [39,40]. We observed that *S*. Enteritidis retrieved from different patients in ILO (in 2019) had highly similar genetic features (antimicrobial resistance determinants, virulence, plasmids replicons) and clustered together at 0–1 SNP distances between them. The isolates were recovered on the 27^th^ of June, 26^th^ of August and 28^th^ of August 2019 and their genetic, geographic and temporal connectedness may be indicative of a previously unrecognized outbreak. Both *S*. Typhimurium ST313 and *S*. Enteritidis ST11 are dominant clones in sub-Saharan Africa [40] and are a major cause of invasive disease, with a corresponding high case-fatality rate [14]. These serovars are justifiably vaccine development priorities. Non typhoidal *Salmonella* serovars, such as Dublin, Infantis, Chester, Rissen have been reported severally from food animals [4,41–44], their presence in human invasive human infection may attest to concerns with water and food safety, including animal contact [45–47]. Although no ARGs were detected in the genomes of these serovar isolates, they remain a public health concern [14,47]. The single occurrence of an acquired quinolone resistance gene, *qnrB19* in this study was detected in *S*. Telelkebir. The strain also expressed phenotypic resistance to the quinolones nalidixic acid and ciprofloxacin. *S*. Telelkebir has been reported a few times from Africa (as seen in Enterobase, [48]), and are more commonly reported in parts of Europe, China and USA [49]. The expansion of atypical *Salmonella* serovars in invasive infections is associated with a high health burden [17,38]. Invasive NTS vaccines in the pipeline may not cover all NTS serovars [50], and we identified several in this study, harbouring an assortment of virulence and antimicrobial resistance determinants. This points to the need for widespread and robust access to invasive *Salmonella* diagnostics in Nigeria to elucidate on the burden and make a case for serovar vaccine priorities [17,38,49].

Amongst a plethora of virulence determinants present on both *S*. Typhi and iNTS, we observed that *S*. Telelkebir, *S*. Durham and *S*. Chester isolates harboured the cytolethal distending toxin islet genes (*cdtB*, *pltA*, *pltB*) also known as typhoid toxin. These toxins were originally thought to be restricted to serovars Typhi and Paratyphi A [51]. However, these have now been reported in other NTS serovars including Bredeney, Javiana, Montevideo, Schwarzengrund, and more recently in Telelkebir [52–54]. A literature search on PubMed and Google Scholar revealed little information on these toxins being reported in *S*. Durham and *S*. Chester. The cytolethal distending toxin islet cause DNA damage and cell cycle arrest in impaired cells [55]. More implicatively, these genes encoded by NTS serovars have been reported to play vital roles in disease pathogenesis [53,54]. Many of *Salmonella* virulence determinants are clustered in pathogenicity island on the bacterial chromosome, playing key roles in disease pathogenesis [56,57]. A variety of SPIs were identified in this study. The SPI-7 which were exclusively detected in *S*. Typhi in this study are known to be large and major backbone constituent of *S*. Typhi, harbouring several virulence determinants including the Vi antigen [58]. Like in this study, the CS54 island and SPI-14 island are more commonly detected NTS [59–62], with scarce reports in *S*. Typhi, and the CS54 island are suggested to have evolved over multiple horizontal transfers [63]. Thus, this study emphasizes on an expanding number of serovars causing invasive infections in the country, and the public health implications therein. Further studies focussed on molecular analysis of gene content of SPIs in invasive *Salmonella* infections could be pertinent in understanding pathogenesis and aid in the advancement of treatment options [64].

## Conclusion

The outcome of our study emphasizes the need for expanded genomic surveillance of invasive *Salmonella* infections in Nigeria as a valuable tool to monitor antibiotic resistance spread and genetic characterization of circulating lineages in Nigeria. Close monitoring of the dominant *S*. Typhi 3.1.1 clone harbouring the IncY plasmid replicon and gyrA_S83Y chromosomal mutation, identified in all the tertiary hospitals in this study, including other serovars is vital, and this may help to establish strategies for empirical treatment and control of spread of antibiotic resistant lineages. Furthermore, our data suggests that introducing typhoid conjugate vaccines, recommended by the World Health Organization for countries like Nigeria that have a high typhoid disease burden, will have a significant impact on health [65]. Development of vaccines which target NTS would be useful in reducing the overall burden of NTS on the continent. Rigorous surveillance plays an essential part in determining which serovars most require coverage, as we observe *S*. Enteritidis to be most prevalent NTS in invasive infections in southwest Nigeria, and hence recommended as vaccine priorities. Importantly, broader protective effects may be achieved by improvements in water, sanitation and hygiene that could interrupt transmission of the causes of typhoid and other invasive salmonellosis.

## Supporting information

S1 Table Quality control and accession information of *Salmonella* genomes sequenced for this study. (XLSX)

S2 Table Epidemiological metadata, antimicrobial susceptibility testing data and genomic characteristics for *Salmonella* isolates characterized in this study. (XLSX)

S3 Table Pairwise single nucleotide polymorphism (SNP) differences for *Salmonella* Typhi genomes sequenced in this study. (XLSX)

S4 Table Pairwise SNP differences for *Salmonella* Enteritidis genomes sequenced in this study. (XLSX)
